# Vertebral cryptococcosis in an immunocompetent patient - a case report

**DOI:** 10.4314/pamj.v8i1.71158

**Published:** 2011-04-10

**Authors:** Bachir Houda, Ammouri Wafa, Tazi Mezalek Zoubida, Adnaoui Mohamed, Aouni Mohamed, Harmouche Hicham

**Affiliations:** 1 Department of Internal medicine, Avicenne University Hospital, Rabat, Morocco

**Keywords:** Cryptococcosis, vertebral, C neoformans, Morocco

## Abstract

We report an unusual case of 70 years old, immunocompetent woman who was diagnosed with vertebral cryptococcosis. The diagnosis was made on the basis of radiological and histological findings. The outcome was favorable under antifungal treatment.

## Introduction

Cryptococcosis is infection with *Cryptococcus neoformans* fungus. *C neoformans* is an opportunistic fungal infection that defines immunosuppression. It can infrequently be seen in immunocompetent patients. Cryptococcosis presents in two forms: pulmonary and cerebromeningeal. Skeletal cryptococcosis is uncommon. There have been only occasional case reports of thoracic vertebral cryptococcosis presenting as a cord compression in an immunocompetent patient [1].We report a case of an unusual presentation of cryptococcal infection in an immunocompetent patient.

## Patient and case report

A 70 years old woman was admitted for paraplegia. Past medical history of the patient revealed bilateral sciatica evolving since July 2009, not alleviated by symptomatic treatment. A month prior admission, the sciatica symptoms worsened towards a gradual onset of paraplegia, prompting the current consultation. No other significant medical history was noted. On admission, body temperature was 37°, heart rate was 75b/mn, and blood pressure 130/60 mmhg. Neurological examination showed spastic paraparesis, deep tendon reflexes of the lower extremities were lively. Babinski reflex was bilaterally positive. Laboratory findings showed: erythrocyte sedimentation rate 100mm/h, white blood cell count 4800/ mm^3^, lymphocytes 1600/mm^3^ and haemoglobin level 12g/dl

HIV serology was negative; complement levels and measurement of CD4/CD8 T-lymphocyte ratio were within the normal range.

**Figure 1:**
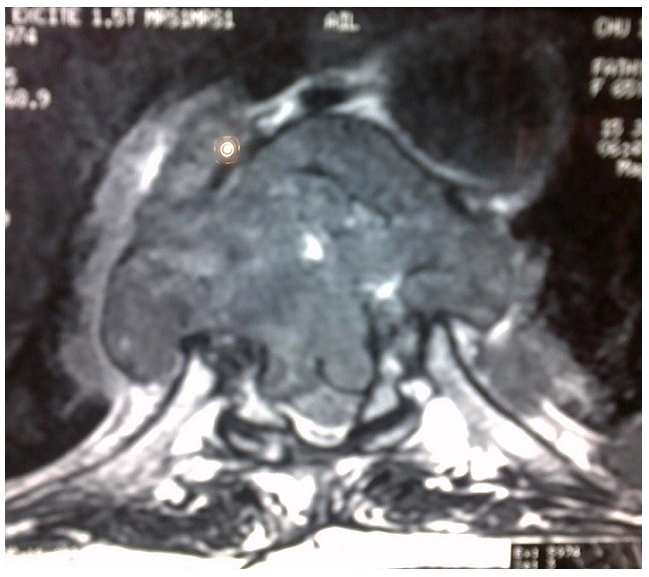
The lesion process extending to the right posterior hemi-arch of the eighth thoracic vertebrae (TH8), responsible for an important spinal cord compression at this level.

**Figure 2:**
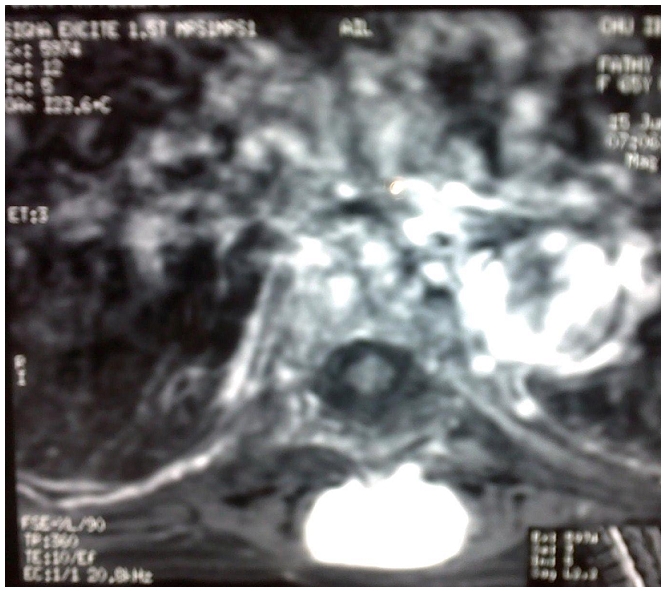
The lesion process extending to the right posterior hemi-arch of the ninth (TH9), responsible for an important spinal cord compression at this level.

A lumbar magnetic resonance imaging was performed showing a lesion process extending from Th8 to Th10, with spinal cord compression. The lesion was hypo-intense on T1-weighted images and hyper-intense on T2-weighted images (Figure 1, 2). Culture of cerebrospinal fluid established the presence of *C neoformans*.

**Figure 3:**
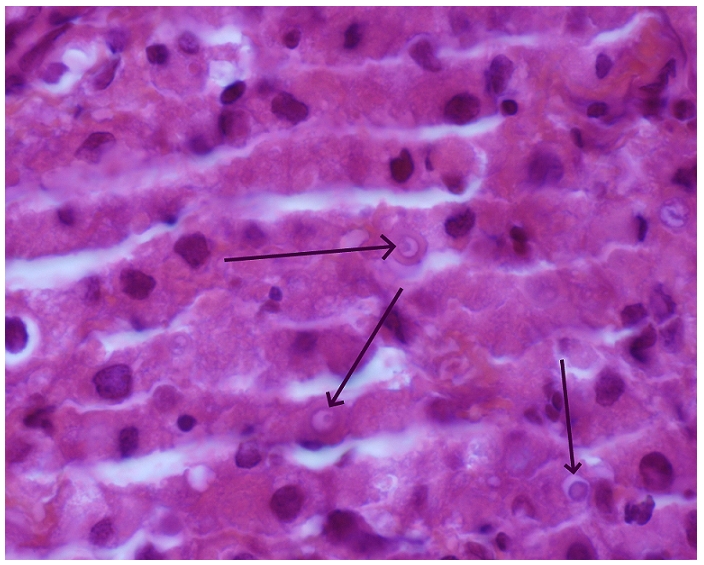
Fungal organisms with thick capsules, a granulomatous inflammatory process without necrosis, an abundant eosinophile cytoplasm and cells with a dark ring in the periphery. Marking cytokeratin was negative (hematoxylin eosin, original × 100)

Posterior laminectomy was performed, revealing an abnormal tissue lesion. Biopsy showed, on histological examination, numerous fungal organisms with thick capsules and a granulomatosis inflammatory process without necrosis. There were no malignant cells (Figure 3). Chest X ray examination and thoracic computed tomography were normal.

The diagnosis of spinal cryptococcosis in an immunocompetent patient was established and the patient was treated by intravenous amphotericin B (0.7 mg/ kg/day) with relay by fluconazol (400mg/d). On follow-up, the patient remains well with no neurological or other sequel.

## Discussion

*Cryptococcus neoformans* is encapsuled yeast, found in pigeon and other bird dropping. It can be inhaled by humans. Most infectious occur in immunocompromised patients, with HIV, transplants patients or patients on long-term corticosteroid therapy [1]. The disease also appears to be more frequent in diabetes [2]. It can rarely be seen in immunocompetent patients [3]. The overall incidence of crytococcosis in immunocompetent individuals has been estimated at 0.2 per million per year [4]. The lung is the portal of entry and then the dissemination is haematogenous [5].

Bones involvement in this Infection is not common. The first described case was reported by Busse and Bushke in 1894 – 1895, which demonstrated that the yeast produced osteomyelitis of the tibia [6]. A variety of bones can be involved by the fungal organism, but vertebras are the single most likely site [7].

The vertebral involvement can mimic tuberculosis of the spine [7]. Like our patient, radiological findings of cryptococcal bone lesions are non-specific, consisting of osteolytic destruction of the vertebral bodies with paraspinal abscesses; thus simulating tuberculosis, which is more prevalent in tropical areas [4].

Diagnosis of bony infection requires biopsy unless *C neoformans*can be is isolated from another body site; less than half of the patients will have a positive serum cryptococcal antigen test [8]. The level of antigen titre correlates with the severity of the disease [5].

Cryptococcal vertebral infection causing cord compression can be treated surgically through debridement and bone grafting. The combination of medical treatment is necessary including, antifungal agents such as amphotericin B, flucytosine and fluconazole [9]. Combination therapy might be needed even if patients are immunocompetent [9].

## Conclusion

Vertebral cryptococcosis can rarely occur in an immunocompetent person without HIV, where it usually goes undiagnosed. The clinician should know this rare and severe infectious complication because the treatment is specific and the prognosis depends of the precociousness of the diagnosis.

## Competing interests

The authors declare no competing interests.

## Authors’ contribution

All authors have read and agreed to the final version of this manuscript and have equally contributed to its content and to the management of the case.
